# Implications for TB control among migrants in large cities in China: a prospective population-based genomic epidemiology study in Shenzhen

**DOI:** 10.1080/22221751.2023.2287119

**Published:** 2023-11-22

**Authors:** Peierdun Mijiti, Changwei Liu, Chuangyue Hong, Meng Li, Xiaoping Tan, Kaiqiao Zheng, Bin Li, Lecai Ji, Qizhi Mao, Qi Jiang, Howard Takiff, Hongxia Fang, Weiguo Tan, Qian Gao

**Affiliations:** a Key Laboratory of Medical Molecular Virology (MOE/NHC/CAMS), School of Basic Medical Science, Shanghai Medical College, Shanghai Institute of Infectious Disease and Biosecurity, Fudan University, Shanghai, People’s Republic of China; b Longhua District Center for Chronic Disease Control, Shenzhen, People’s Republic of China; c Shenzhen Center for Chronic Disease Control, Shenzhen, People’s Republic of China; d Laboratorio de Genética Molecular, CMBC, IVIC, Caracas, Venezuela; e Xinjiang Medical University, School of Public Health, Department of Epidemiology, Wulumuqi, People's Republic of China

**Keywords:** Tuberculosis, transmission, internal migrants, whole-genome sequencing

## Abstract

Internal migrants are a challenge for TB control in large Chinese cities and understanding this epidemiology is crucial for designing effective control and prevention strategies. We conducted a prospective genomic epidemiological study of culture-positive TB patients diagnosed between June 1, 2018 and May 31, 2021 in the Longhua District of Shenzhen. Treatment status was obtained from local and national TB registries and all isolates were sequenced. Genomic clusters were defined as strains differing by ≤12 SNPs. Risk factors for clustering were identified with multivariable analysis and then Bayesian models and TransPhylo were used to infer the timing of transmission within clusters. Of the 2277 culture-positive patients, 70.1% (1596/2277) were migrants: 72.1% (1043/1446) of the migrants patients developed TB within two years of arriving in Longhua; 38.8% within 6 months of arriving; and 12.3% (104/843) had TB symptoms when they arrived. Only 15.4% of Longhua strains were in genomic clusters. More than one third (33.6%) of patients were not treated in Shenzhen but were involved in nearly one third of the recent transmission events. Clustering was associated with migrants not treated in Shenzhen, males, and teachers/trainers. TB in Longhua is prinicipally due to reactivation of infections in migrants, but a proportion may have had clinical or incipient TB upon arrival in the district. Patients diagnosed but not treated in Longhua were involved in recent local TB transmission. Controlling TB in Shenzhen will require strategies to comprehensively diagnose and treat active TB in the internal migrant population.

## Introduction

Tuberculosis (TB) is a leading cause of death worldwide. In China, one of the countries with the largest TB burden, there were an estimated 842,000 new TB cases in 2020, accounting for 8.5% of TB incident cases worldwide [1]. While the overall incidence of TB in China has decreased over the past few decades, the incidence among internal migrants has almost doubled [2]. Currently, about 20% of the total population of China are internal migrants, defined as people, mostly from rural areas, who migrate to the rapidly growing Chinese cities for job opportunities but do not have an urban household registration or residence permit. In many large Chinese cities such as Shenzhen, Shanghai and Guangzhou, internal migrants account for nearly half of the total population [3]. In two of the largest cities, Shanghai and Shenzhen, more than 70% of new TB cases are internal migrants, who have an incidence of TB that is significantly higher than in local residents [4, 5]. This could be related to the difficult living conditions the internal migrants often experience after coming to the cities [6], but perhaps also to the socio-economic conditions in their hometowns that prompted their migration. The incidence of TB in the rural areas of China is higher than in most big cities in China [7].

Whole-genome sequencing (WGS) has helped to elucidate transmission patterns of TB among immigrants in many countries [8–10]. Walker et al. used WGS to investigate the epidemiology of TB transmission in Oxfordshire, UK, 2007-2012, and concluded that the clustering rate was only 15.8%, suggesting that most patients born in high-incidence countries reactivate latent infections acquired before emigrating [8]. A national study conducted in Switzerland during 2000–2008 obtained similar results and determined that the clustering rate among immigrants was only 6.5% [9]. We previously used WGS to show that TB cases in large Chinese cities are predominantly the result of reactivation of latent TB infections that the internal migrants acquired in their hometowns and brought with them to the cities [11, 12].

In Shenzhen, which has grown from 30,000 inhabitants in 1978 to approximately 17 million today, internal migrants comprise over 70% of the total population and more than 90% of the registered TB cases [6]. In our previous genomic epidemiological study we retrospectively investigated the source of TB cases diagnosed and treated in the Bao’an District of Shenzhen [11]. To further investigate the TB epidemiology and foster targeted TB control strategies in Shenzhen and other Chinese cities, we performed a prospective population-based cohort study of all culture-positive patients diagnosed in the Longhua District of Shenzhen from 2018 to 2021.

## Methods

### Study setting

The Longhua District is located in the northwest region of Shenzhen and has a total population of about 2,500,000. Of Shenzhen’s 10 administrative districts, Longhua has the highest concentration of industrial parks, which attract the migrant workers who constitute over 75% of the district’s population [13]. In 2018-2020, The average TB case notification rate in Longhua was 41.4 per 100,000 populations.

### Study participants and data collection

The Longhua District Center for Chronic Disease Control (CCDC) is responsible for the diagnosis and treatment of pulmonary TB (PTB) in the district. We conducted a genomic epidemiology study with prospective collection of clinical isolates. Sputum samples for microscopic examination and culture were obtained from all individuals with abnormal chest radiographs who visited the Longhua District CCDC TB clinic from June 1, 2018 to May 31, 2021. To avoid losing patients to follow up, an epidemiologic investigation was conducted at the time of sputum collection to obtain demographic and clinical data. When patients were diagnosed with TB, they were contacted by health care workers to ascertain whether they would receive treatment in Shenzhen or would return to their hometowns for treatment. Regardless of whether they received anti-TB treatment in Shenzhen or in their hometowns, all culture-positive patients aged ≥15 years were included in this study.

In this study, all patients with Shenzhen household registrations or residence permits were defined as residents. Individuals with residence permit generally have had a fixed residence in Shenzhen for at least 12 months and a stable job for at least 18 months. Those without a Shenzhen household registration or residence permit were defined as internal migrants.

### WGS

The DNA from all clinical isolates obtained during the study period was extracted with the cetyltrimethylammonium bromide (CTAB) method and sequenced on an Illumina HiSeq 2500 platform with an expected depth of 100x, as previously described [11, 14]. Raw sequence data were uploaded to the online platform SAM-TB (https://samtb.uni-medica.com) for genomic analysis [15]. In brief, raw sequence reads were trimmed with Sickle to remove reads of low quality (Phred base quality < 20 or read length < 35) and aligned to the reference genome (H37Rv, NC000962.3) using BWA (version 0.7.15). SAMtools and Varscan were then applied to the alignments to call single nucleotide polymorphisms (SNPs). The fixed SNPs (frequency ≥75%), excluding those in drug-resistance associated genes and repetitive regions of the genome (e.g. PPE/PE-PGRS family genes, phage sequences, insertion or mobile genetic elements), were used to calculate the pairwise SNP distances. Strains with a sequence depth less than 20x or a genome coverage less than 95% were excluded from the analysis. A genomic cluster was defined as strains differing by 12 or fewer SNPs [11]. Phylogenetic trees based on the identified SNPs were constructed using RAxML-NG software with the maximum likelihood method and 100 bootstraps, and visualized with Interactive Tree of Life (https://itol.embl.de/). Pan-susceptible TB was defined as susceptibility to the four first-line drugs (isoniazid, rifampicin, ethambutol and pyrazinamide). Multidrug-resistant TB (MDR-TB) was defined as resistance to at least isoniazid and rifampicin. Strains with resistance to any of the four first-line drugs but not MDR were termed other drug resistant (DR) TB. Pre-XDR was defined as MDR with additional resistance to the fluoroquinolones.

### Transmission inference

Transmission inference was performed as previously described [12]. In brief, a Bayesian evolutionary analysis was used to infer a time-labeled phylogeny by sampling trees with BEAST (version 1.8.4), using a Markov chain Monte Carlo approach with TransPhylo (version 1.4.5) to estimate infection times and putative direction of transmission between clustered patients [12, 16, 17]. We quantified the contribution of transmission from TB patients not enrolled in treatment in Shenzhen by comparing the estimated infection time of the secondary case with the diagnostic date of the index case. Transmission was considered to have occurred from these cases if the infection time of the secondary case was after the date on which the index case was diagnosed.

### Epidemiological investigation

A questionnaire administered to all culture-positive TB patients within a week of their diagnosis asked about the patients’ close contacts, workplaces, residential addresses, and social settings frequented in the three years prior to their TB diagnosis. After WGS, patients whose clinical strains belonged to genomic clusters were invited to participate in an in-depth interview to explore epidemiological links with other patients in the same cluster. The epidemiologic links were defined as confirmed when clustered patients knew each other and had a history of contact before TB diagnosis.

### Statistical and spatial analysis

Statistical analysis was performed in Stata 14.0, using the chi-square test to compare categorical variables and the Wilcoxon rank sum test for non-normal distributed continuous variables. Univariable and multivariable logistic regression were performed to identify factors associated with genomic clustering and separately, being enrolled in treatment in Shenzhen. The multivariable regression used backward selection on variables with *P*-values less than 0.2 in the univariate analysis to obtain crude and adjusted odds ratios with 95% confidence intervals. *P*-values <0.05 were considered significant. Spatial analysis and visualization were performed in ArcGIS (version 10.2). Point data was used for mapping the cases by kernel density estimation, using Gaussian smoothing to analyze patient aggregation [18].

## Results

### Characteristics of the study population

**Figure 1.**
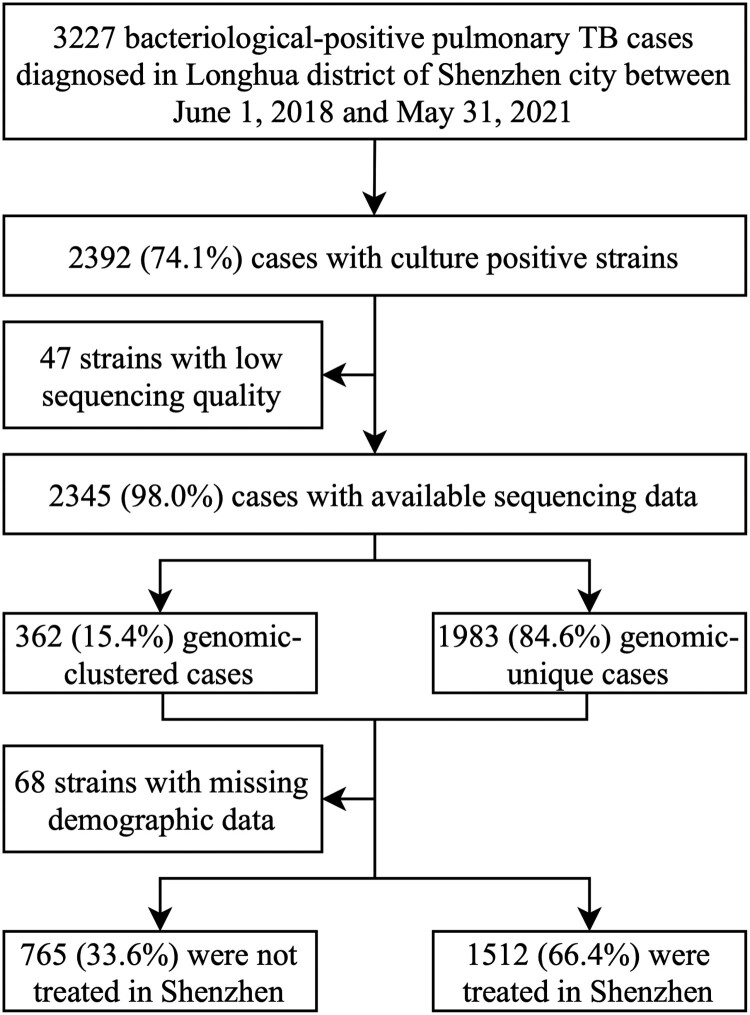
Sample enrolment and study flow chart.

A total of 3227 bacteriological-positive TB patients were diagnosed in the Longhua District CDCC between June 1, 2018, and May 31, 2021, of whom 2392 (74.1%) were culture-positive. After excluding 47 strains with low sequencing quality and 68 strains without clinical and demographic data, a total of 2277 PTB patients were included in the subsequent analysis (Figure 1). The mean age of these 2277 patients was 32.2 years (range 15–83 years), and the majority were males, aged 15–34 years, employed in housekeeping or unemployed, with no previous history of TB. Nearly half (47.1%, 1071/2277) lived in the Guanlan and Longhua sub-districts, where most of the district’s industrial parks are located (Table 1).

**Table 1.** Characteristics of PTB cases diagnosed in Longhua District of Shenzhen between June 1, 2018 and May 31, 2021.

	Total (n = 2277)	Residents (n = 681)	Migrants (n = 1596)	*P* values
**Demographic characteristics**				
Gender				
Male	1646 (72.3)	449 (65.9)	1197 (75.0)	<0.001
Female	631 (27.7)	232 (34.1)	399 (25.0)	
Age (years)				
15–34	1614 (70.9)	415 (61.0)	1199 (75.1)	<0.001
35–54	500 (22.0)	219 (32.2)	281 (17.6)	
55–64	114 (5.0)	36 (5.3)	78 (4.9)	
65 and higher	49 (2.1)	11 (1.6)	38 (2.4)	
Occupation				
Housekeeping/jobless	864 (37.9)	205 (30.1)	659 (41.3)	<0.001
Factory workers	811 (35.6)	257 (37.7)	554 (34.7)	
Students	59 (2.6)	14 (2.1)	45 (2.8)	
Teachers/tutors	19 (0.8)	9 (1.3)	10 (0.6)	
Others	524 (23.0)	196 (28.8)	328 (20.6)	
Residence time in Longhua (months) (n = 2091)^†^				
Median (IQR)	24 (5-60)	60 (24-120)	12 (2-36)	<0.001
0–6	623 (29.8)	62 (9.6)	561 (38.8)	<0.001
7–23	629 (30.1)	147 (22.8)	482 (33.3)	
24–59	398 (19.0)	168 (26.0)	230 (15.9)	
60 and higher	441 (21.1)	268 (41.6)	173 (12.0)	
Living address at diagnosis				
Longhua subdistrict	592 (26.0)	190 (27.9)	402 (25.2)	0.057
Guanlan subdistrict	479 (21.1)	126 (18.5)	353 (22.1)	
Other subdistricts	971 (42.6)	305 (44.8)	666 (41.7)	
Othe districts	235 (10.3)	60 (8.8)	175 (11.0)	
**Clinical characteristics**				
Retreated cases	104 (4.6)	32 (4.7)	72 (4.5)	0.844
Sputum smear-positive	767 (33.7)	234 (34.4)	533 (33.4)	0.655
Cavitary disease	598 (26.3)	178 (26.1)	420 (26.3)	0.930
Diagnostic delay (n = 1495)*				
<2 weeks	547 (36.6)	196 (34.5)	351 (37.9)	0.033
2–4 weeks	314 (21.0)	140 (24.6)	174 (18.8)	
4–8 weeks	350 (23.4)	136 (23.9)	214 (23.1)	
>8 weeks	284 (19.0)	97 (17.1)	187 (20.2)	
Reported having TB symptoms before arrival (n = 1495)*				<0.001
Yes	125 (8.4%)	11 (1.9)	114 (12.3)	
No	1370 (91.6)	563 (98.1)	807 (87.7)	
**Bacteriological characteristics**				
Genotype				
Beijing strain	1693 (74.4)	507 (74.5)	1186 (74.3)	0.946
Non-Beijing strain	584 (25.7)	174 (25.6)	410 (25.7)	
Drug-resistance profile				
MDR	99 (4.3)	23 (3.4)	76 (4.8)	0.311
Other DR	325 (14.3)	101 (14.8)	224 (14.0)	
Pan-susceptible	1853 (81.4)	557 (81.8)	1269 (81.2)	

TB, tuberculosis; PTB, pulmonary TB; MDR, multi-drug resistance; DR, drug resistance; IQR, interquartile range

^†^ Residence time was uknown for 186 patients

*Dates of symptoms onset were unknown for 782 patients

### Comparison of the demographic and clinical characteristics of internal migrant and resident TB patients

Of the patients included in the study, 70.1% (1596/2277) were internal migrants. Although the clinical characteristics were similar between migrant and resident TB patients, migrant patients were more likely to be male, aged 15–34 years and unemployed or working in housekeeping (Table 1).

**Figure 2.**
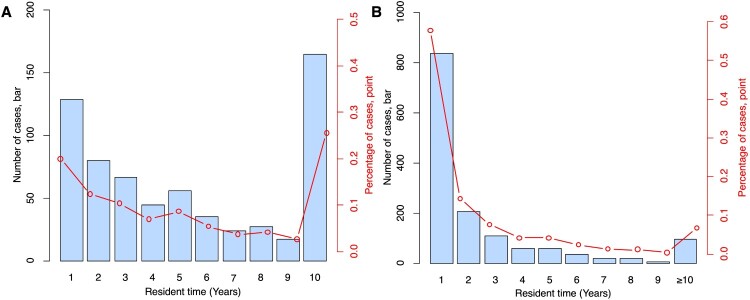
The number (bars) and proportion (points) of resident (A) and migrant (B) TB patients by years of residence time in the Longhua district.

To investigate how soon after arriving in Longhua the migrants developed TB, we used the length of time the patients had resided at the address given at the time of diagnosis. Among the 681 resident patients, 574 (84.3%) had moved from neighbouring districts of Shenzhen to the Longhua District. For these patients, the time they resided in Longhua was taken as the time since they left the neighbouring districts until their TB diagnosis. The median time from arriving in Longhua to TB diagnosis was significantly shorter for internal migrants than for residents (12 months vs. 60 months, *P* < 0.01). Most cases of TB in internal migrants (57.8%, 836/1446) were diagnosed during the first year after arriving in Longhua, with 38.8% diagnosed within the first 6 months. Nearly two thirds (72.1%, 1043/1446) of migrant patients were diagnosed within the first 2 years, and the number of cases declined progressively as the years of residence in Longhua increased (Figure 2). Among the 921 internal migrant TB patients who reported dates of arrival and onset of TB symptoms (cough, haemoptysis, fever, night sweats, weight loss), 12.3% (114/921) reported having at least one of these symptoms when they arrived in Longhua, and 90.3% (72/114) of these were diagnosed within two months after arriving. By comparison, among 645 resident TB patients who reported dates of arrival, just 32.4% (209/645) were diagnosed within two years after arriving, and among 574 patients who reported dates of onset of TB symptoms, only 1.9% (11/574) had at least one TB symptom when they arrived in Longhua (Figure 2).

### WGS for genotyping and drug resistance prediction

**Figure 3.**
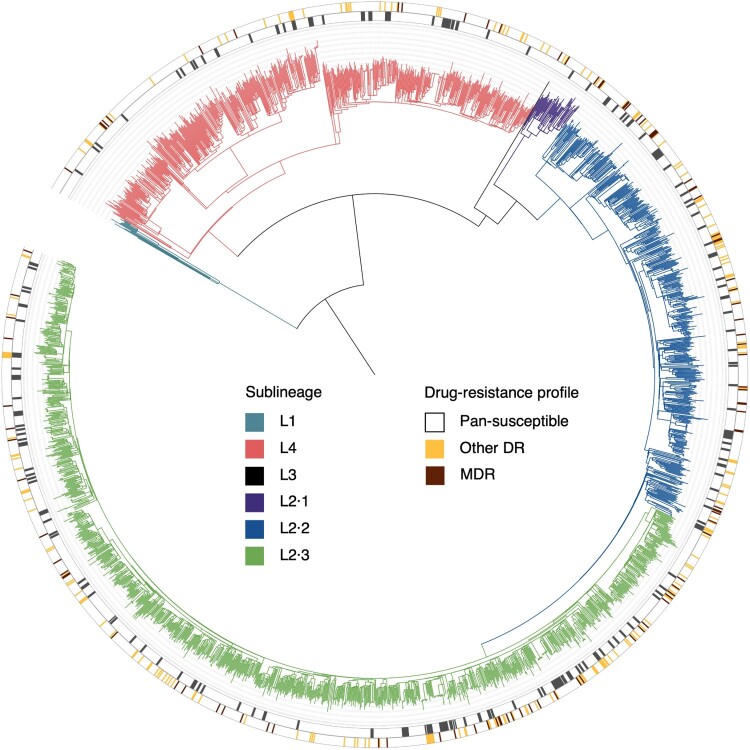
Phylogeny, clustering, and resistance profile of 2345 Mycobacterium tuberculosis strains isolated in the Longhua district during June, 2018-May, 2021. The different colours on the branches indicate different lineages and sublineages. The outer grey circle indicates genomic-clustered strains differing by ≤12 single-nucleotide polymorphisms. The outer yellow-brown circle indicates other drug resistance and multidrug resistance.

Phylogenetic analysis of the WGS from the 2345 TB strains, including the 68 strains with missing demographic and clinical data, showed that 74.1% (1737/2345) (Figure 3) belonged to L2 with 67.1% (1164/1737) modern and 32.9% (572/1737) ancient Beijing sub-lineages. L4 strains constituted 25.4% (596/2345) of the total strains, L1 strains were 0.5% (12/ 2345) and there was one L3 strain.

Analysis of genome sequences for mutations conferring resistance to 14 anti-TB drugs showed that 86.0% (2017/2345) of the strains were pan-susceptible and 14.0% (328/2345) were resistant to at least one first-line anti-TB drug. Mutations conferring resistance to isoniazid were present in 10.1% (237/2345), mutations conferring resistance to rifampicin were present in 7.6% (178/2345), and resistance to both (MDR-TB) was observed in 4.4% (103/2345). There was greater percentage of every type of drug resistance in retreated cases compared to new cases (Table 2), but there was no statistical difference in resistance between strains from internal migrants and those from residents (Supplementary Table S1).

**Table 2.** Drug-resistance profile, stratified by new and retreated cases.

	New cases (n = 2173)	Retreated cases (n = 104)	Total (n = 2277)
Pan-susceptible	1884 (85.7)	75 (72.1)	1959 (86.0)
Any resistance to INH	206 (9.5)	25 (24.0)	231 (10.1)
Any resistance to RIF	151 (7.0)	23 (22.1)	174 (7.6)
Any resistance to EMB	53 (2.4)	13 (12.5)	66 (2.9)
Any resistance to PZA	41 (1.9)	4 (3.9)	45 (2.0)
MDR	80 (3.7)	19 (18.3)	99 (4.4)
MDR plus resistance to any FQ	21 (1.0)	5 (4.8)	26 (1.1)

INH, isoniazid; RIF, rifampicin; EMB, ethambutol; PZA, pyrazinamide; MDR, multidrug resistance; FQ, fluoroquinolone

### Cluster identification and associated risk factors

Analysis of the WGS of the 2345 strains revealed that 362 (15.4%, 362/2345) belonged to 139 genomic clusters containing 2–10 strains each (Figure 2). After excluding the 68 strains without clinical and demographic information, we analyzed the data from 2277 TB cases to identify factors associated with genomic clustering. Multivariable logistic regression revealed that males, age less than 55 years, teachers/tutors, Beijing genotype, migrants not enrolled in treatment in Shenzhen, and residence time in Longhua >6 months were associated with genomic clustering. Interestingly, MDR-TB cases were less likely to be in genomic clusters than drug sensitive strains or strains with other resistance profiles (Table 3).

**Table 3.** Univariate and multivariable logistic regression of risk factors for clustering.

	Clustered (n = 351)	Non-clustered (n = 1926)	Univariate analysis		Multivariable analysis	
			cORs (95% CI)	*P* values	aORs (95% CI)	*P* values
Gender						
Male	271 (77.2)	1375 (71.4)	1.35 (1.04-1.78)	0.026	1.43 (1.08-1.89)	0.012
Female	80 (22.8)	551 (28.6)	1.00		1.00	
Age (years)						
15–34	276 (78.6)	1374 (71.3)	2.67 (1.38-5.15)	0.003	2.91 (1.54-5.48)	0.001
35–54	65 (18.5)	419 (21.8)	2.06 (1.03-4.13)	0.041	2.26 (1.16-4.42)	0.017
≥55	10 (2.9)	133 (6.9)	1.00		1.00	
Occupation						
Teachers/tutors	7 (2.0)	12 (0.6)	3.25 (1.27-8.30)	0.014	3.03 (1.15-7.97)	0.024
Others	344 (98.0)	1914 (99.4)	1.00		1.00	
Status of being enrolled in treatment in Shenzhen stratified by residency						
Migrants not enrolled in treatment	123 (35.0)	535 (27.8)	1.55 (1.22-1.87)	0.013	1.64 (1.25-2.14)	<0.001
Residents not enrolled in treatment	18 (5.1)	89 (4.6)	1.37 (0.80-2.33)	0.240	1.34 (0.78-2.31)	0.283
Those enrolled in treatment	210 (55.8)	1302 (67.6)	1.00		1.00	
Residence time in Longhua (months)						
≤6 months	76 (21.7)	547 (28.4)	1.00		1.00	
>6 months	250 (71.2)	1218 (63.2)	1.48 (1.12-1.95)	0.006	1.89 (1.40-2.53)	<0.001
Unknown (n = 186)	25 (7.1)	161 (8.4)	1.12 (0.69-1.81)	0.653	1.11 (0.68-1.84)	0.655
Sputum smear-positive						
Yes	130 (37.0)	637 (33.1)	1.19 (0.94-1.51)	0.148	-	
No	221 (63.0)	1289 (66.9)	1.00		-	
Cavitary disease						
Yes	98 (28.0)	500 (26.0)	1.10 (0.85-1.42)	0.443	-	
No	253 (72.0)	1426 (74.0)	1.00		-	
Beijing strain						
Yes	288 (82.1)	1405 (73.0)	1.70 (1.27-2.27)	<0.001	1.64 (1.21-2.21)	0.001
No	63 (18.0)	521 (27.1)	1.00		1.00	
MDR-TB						
Yes	7 (2.0)	92 (4.8)	0.41 (0.19-0.88)	0.023	0.37 (0.17-0.81)	0.013
No	344 (98.0)	1834 (95.2)	1.00		1.00	

cOR, crude odds ratios; aOR, adjusted odds ratio; MDR, multi-drug resistance; DR, drug resistance

### Characteristics of TB cases not enrolled in treatment in Shenzhen and their contribution to recent local TB transmission

A total of 765 (33.6%, 765/2277) TB patients diagnosed in Longhua were not registered for treatment in Shenzhen. Of these 765 cases, 337 (44.1%) were lost to follow-up. The remaining 428 (55.9%) cases were documented to have returned to their hometowns or other places where they were registered as having received TB treatment.

The majority of the TB cases diagnosed but not treated in Shenzhen were male (78.8%, 603/765), internal migrants (86.0%, 658/765), aged 15–34 years (77.8%, 595/765), who had negative sputum-smears (73.9%, 565/765) (Table 4). Multivariable analysis revealed that all of these characteristics were significantly associated with not being treated in Shenzhen. Patients residing ≤ 6 months in Longhua were also less likely to be treated in Shenzhen compared to those who had lived in Longhua >6 months. An initial analysis showed that factory workers were more likely to be treated in Shenzhen than patients who were unemployed or working in housekeeping. However, subgroup analyses revealed that only resident factory workers, but not migrant factory workers, were more likely to be treated in Shenzhen (Supplementary Table S2 and Table S3).

**Table 4.** Characteristics of cases enrolled and not enrolled in treatment in Shenzhen.

	Enrolled in treatment in Shenzhen		*P* values	Multivariable analysis*	
	Yes (n = 1512)	No (n = 765)		aORs (95%CI)	*P* values
**Demographic characteristics**					
Gender					
Male	1043 (69.0)	603 (78.8)	<0.001	1.58 (1.26-1.99)	<0.001
Female	469 (31.0)	162 (21.2)		1.00	
Age (years)					
15–34	1019 (67.4)	595 (77.8)	<0.001	1.00	
35–54	389 (25.7)	111 (14.5)		0.64 (0.49-0.83)	0.001
55–64	73 (4.8)	41 (5.4)		1.18 (0.76-1.83)	0.454
65 and higher	31 (2.1)	18 (2.4)		0.89 (0.46-1.73)	0.725
Occupation					
Housekeeping/jobless	533 (35.3)	331 (43.3)	<0.001	1.00	
Factory workers	587 (38.8)	224 (29.3)		0.78 (0.62-0.98)	0.034
Students	32 (2.1)	27 (3.5)		1.24 (0.69-2.23)	0.467
Teachers or tutors	12 (0.8)	7 (0.9)		1.78 (0.64-4.98)	0.271
Others	348 (23.0)	176 (23.0)		1.13 (0.88-1.46)	0.346
Time to diagnosis since arrival at Longhua district (months)					
Median (IQR)	24 (12-72)	8 (1-24)	<0.001		
≤6 months	309 (20.4)	314 (41.0)	<0.001	2.66 (2.15-3.30)	<0.001
>6 months	1150 (76.1)	318 (41.6)		1.00	
Unknown (n = 186)	53 (3.5)	133 (17.4)		8.57 (5.99-12.28)	<0.001
Residency status					
Residents	574 (38.0)	107 (14.0)	<0.001	1.00	
Migrants	938 (63.0)	658 (86.0)		2.73 (1.82-3.01)	<0.001
**Clinical characteristics**					
Retreated cases	68 (4.5)	36 (4.7)	0.822		
Cavitary disease	404 (26.7)	194 (25.4)	0.486		
Sputum-smear positivity					
Positive	567 (37.5)	200 (26.1)	<0.001	1.00	
Negative	945 (62.5)	565 (73.9)		1.67 (1.34-2.07)	<0.001
**Bacteriological characteristics**					
Beijing strain					
Yes	1132 (74.9)	561 (73.3)	0.428		
No	380 (25.1)	204 (26.7)			
Drug-resistance profile					
MDR	58 (3.8)	41 (5.4)	0.113		
Other DR	204 (13.5)	116 (15.2)			
Pan-susceptible	1250 (82.7)	608 (79.5)			

PTB, pulmonary tuberculosis; cOR, crude odds ratios; aOR, adjusted odds ratio; MDR, multi-drug resistance; DR, drug resistance

We then tried to determine whether the patients who were not treated in Shenzhen had contributed to recent TB transmission in Longhua. Of clustered cases, 30.8% (108/351) were not treated in Shenzhen, and 55.4% of genomic clusters (77/139) contained at least one TB case not treated in Shenzhen. We then used the Bayesian approach with TransPhylo to infer the time of infection in 11 clusters with at least 4 patients. After excluding the initial transmission to the putative source case of each cluster, we estimated 69 transmission events occurring among 69 genomic clustered cases, and in 53 of these, both patients were captured in the study (Supplementary Fig. S1 and S2). Out of these 53 transmission events, 39.6% (21/53) were initiated by TB cases not treated in Shenzhen, and of these, 33.3% (7/21) of transmission events occurred after the transmitting case was diagnosed. Further analysis revealed that 4 of these 7 were lost to follow-up and the other 3 returned to their hometowns, but with delays of 52–120 days between diagnosis in Shenzhen and initiation of TB treatment in their hometowns.

### Epidemiological investigation

To further delineate the transmission links between the genomic-clustered patients, we performed in-depth epidemiological phone interviews of the clustered patients. Epidemiological links were identified in just 18 (5.3%, 18/341) clustered cases, including 4 pairs of family members, 3 teacher-student pairs and 2 cohabiting couples.

### Spatial distribution of clustered and unclustered TB cases

**Figure 4.**
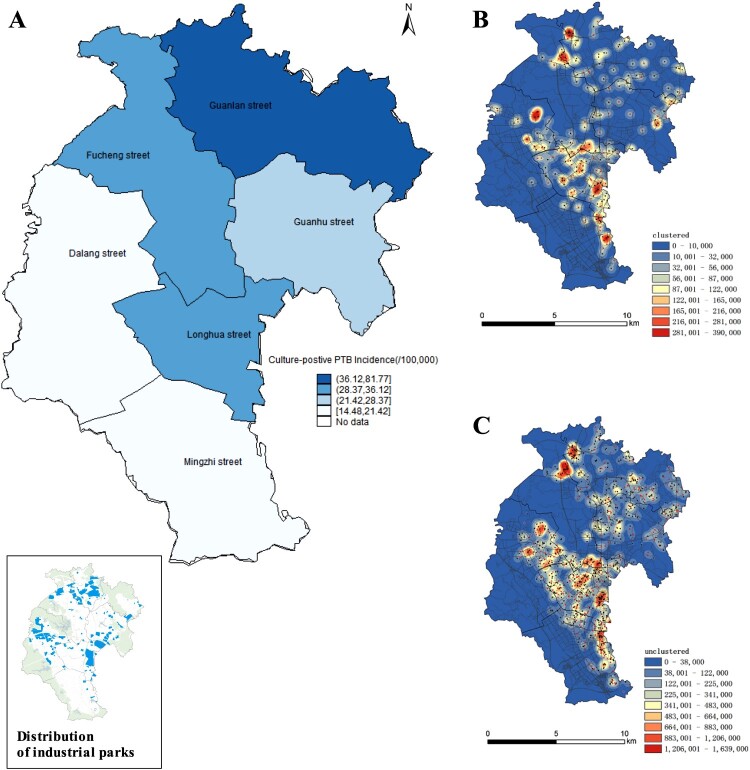
Spatial distribution TB cases diagnosed in Longhua district. (A) incidence rates for culture-positive pulmonary TB in Longhua sub-districts. Insert in (A), location of industrial parks in Longhua district. Kernel density estimation (KDE) for clustered (B) and non-clustered pulmonary TB cases (C) diagnosed in Longhua district between June 1, 2018 and May 31, 2021. Red dots indicates resident patients and black dot indicates migrant patients in B and C.

To characterize the spatial distribution of the cases, we used the residential address of the patients (n = 2198) to draw kernel density maps to locate clustered (Figure 4b) and unclustered TB cases (Figure 4c). The incidence of culture-positive TB was highest in the Guanlan sub-district, followed by the Fucheng and Longhua sub-districts, all of which contain industrial parks (Figure 4a). Hotspots with high concentrations of both clustered and unclustered TB cases were mainly in communities or urban villages surrounding the industrial zones (Figure 4). The median geographic distance between the residences of all the 365 paired clustered cases was 3.33 (ranges 1.19-6.81) kilometres.

## Discussion

This prospective genomic epidemiological study revealed that only 15.4% of the culture-positive TB patients in Longhua District of Shenzhen were in genomic clusters. Our results show that 70.1% of TB patients in Longhua were rural-to-urban migrants, 65.4% of whom had TB diagnosed within two years of arrival, and 12.3% reported having TB symptoms when they arrived in Longhua. Of the TB cases diagnosed in Longhua, 33.6% were not treated in Shenzhen and 86% of these were internal migrants. Nearly one fifth of the TB patients who were not treated in Shenzhen belonged to clusters and were involved in recent transmission, perhaps due to delayed return to their hometowns or being lost to follow-up and untreated.

It is therefore important to determine why 33.6% of the culture-positive patients diagnosed in Longhua were not treated in Shenzhen. The majority of the TB cases diagnosed but not treated in Shenzhen were internal migrants (86.0%). Unlike immigrants in low-incidence countries, the rural-to-urban internal migrants in China maintain strong ties with their hometowns. When they are diagnosed with TB in the city, many choose to return to their hometowns for treatment because they are unemployed or work in housekeeping (41%) and lack health insurance. Some of those diagnosed with TB were lost to follow-up because they didn’t give accurate contact or had left the district [18–20]. In recent years, China has tried to address the management of migrant TB patients in urban settings by establishing a national TB registry to trace TB diagnosed cases. There has also been an effort to strengthen the cooperation and communication between urban and rural TB control programmes to ensure timely initiation of treatment and follow-up [21]. However, our results suggest that the level of this communication may need to be upgraded to ensure the timely treatment of all diagnosed patients.

Analysis of the characteristics of the TB cases who were not treated in Shenzhen revealed that the middle aged patients (35-54 years) and resident factory workers were more likely to seek treatment in Shenzhen, perhaps indicating greater access to TB education and healthcare facilities. Migrant factory workers, however, were not more likely to be treated in Shenzhen, indicating a need to improve health education and medical coverage for this population to insure that they receive prompt anti-TB treatment to minimize transmission.

Patients who are diagnosed with TB in a Chinese city but not enrolled in treatment in the city, likely contribute to local transmission. A molecular epidemiological study of 11 community clusters in the UK revealed that TB patients who were not enrolled promptly in treatment or who did not comply with treatment often became super-spreaders for community outbreaks [22]. In this study, we showed that not being treated in Shenzhen was associated wth clustering, suggesting that some of the TB patients were transmitting their infections in Longhua before returning to their hometowns. Only a third of the TB patients in this study were positive on initial sputum microscopy (Table 1) and many were diagnosed only several weeks later when their sputum cultures became positive. The implementation of same-day molecular tests with better sensitivity than sputum smears would reduce the frequent delays from the time of diagnosis to the initiation of treatment and thereby decrease the opportunities for transmission. More TB patients could be diagnosed at their initial visits and enrolled in TB treatment immediately. Once on treatment, patients wishing to leave Shenzhen could continue their treatment uninterrupted after the medical institutions in Shenzhen communicate with the TB programmes in the hometowns.

The genomic clustering rate in Longhua, Shenzhen was only 15.4%, similar to clustering rates in countries with low TB burdens, such as the UK (15.8%) [7] and Switzerland (6.5%) [8]. The findings in the current study are also consistent with our results from a similar study conducted in the Bao’an District of Shenzhen (12.2%) [11]. Both studies determined that the burden of tuberculosis was largely due to the reactivation of latent infections rather than local transmission. Our prospective study in Longhua, however, suggests that a large number of migrants may have arrived with active or incipient TB, because 38.8% were diagnosed within 6 months of arriving in Longhua and 12.3% of the patients reported they had tuberculosis symptoms when they arrived. This highlights the importance of TB screening in the migrant population. Many countries with a low TB burden conduct screening for active TB among immigrants from high TB burden countries [23–25]. Screening for active TB in the United States [26] and the United Kingdom [27] reduced the incidence of TB cases in the first year after immigration by 40%. Similarly, screening migrants arriving Shenzhen for active TB could reduce the TB burden, but would be challenging. A possible strategy might entail screening migrants entering employment, but this could miss nearly half of migrant TB patients who are either unemployed or work at informal housekeeping jobs. Despite these obvious difficulties, it seems clear that reducing the incidence and spread of TB in Shenzhen will require screening strategies to find and treat TB in arriving internal migrants, and then periodic screening to detect new cases in this population, at least through their first two years after arrival.

Although the level of recent transmission in Longhua District was very low, we also identified the same high-risk groups identified in previous studies [11, 12]. Because of the greater mobility of males and young people, these populations were at higher risk of recent transmission. Teachers/tutors also had a relatively higher risk of recent transmission, although this may be largely attributable to a TB outbreak in an educational institution that occurred during the course of the study. Educational institutions are densely populated settings where outbreaks of tuberculosis have been frequently reported [28–30]. Therefore, there is a need to strengthen TB screening and management in educational institutions.

This study had some limitations. It only included TB patients diagnosed in Longhua, but transmission can also occur between individuals living in Longhua and those living in the other nine districts of Shenzhen. To demonstrate cross district transmission, we compared the WGS data of Longhua strains with the WGS of strains isolated in the Bao’an District from 2014 to 2017 [11], and identified 7 clusters containing patients from both districts. Another limitation is that the duration of this study was only 3 years, ending in May of 2021. It thus included almost 18 months when pandemic measures to restrict the spread of Covid-19 could have reduced TB diagnosis and resulted in an underestimation of tuberculosis transmission. In addition, the high mobility of migrant TB patients and the low percentage contacted for detailed epidemiological telephone surveys could have affected the accuracy of the epidemiological statistics.

In conclusion, new TB cases in the Longhua District of Shenzhen are mainly caused by the reactivation of latent infections in the migrant population, most of whom develop TB within two years after arriving in the district. A smaller, but significant group probably had active or incipient TB when they arrived in Longhua. Nearly one third of the patients diagnosed in Longhua were not treated in Shenzhen and some of these belonged to transmission clusters. Control of TB in Shenzhen will require new strategies to comprehensively find and treat active infections in the migrant population. More rapid and effective diagnostic methods and increased coordination with rural, hometown TB programmes would decrease the chances for transmission and the reduce the number of TB diagnosed patients lost to follow-up.

## Supplementary Material

Supplementary_material_revised

## Data Availability

Sequencing data were deposited in the Genome Sequence Archive (https://bigd.big.ac.cn/gsa) under BioProject PRJCA013222. De-identified participant data from the study will be made available upon publication to medical researchers on a not-for-profit basis by email request to the corresponding author for the purposes of propensity matching or meta-analysis.
