# Supplementary material for: Implications for TB control among migrants in large cities in China: a prospective population-based genomic epidemiology study in Shenzhen
Source: Emerg Microbes Infect. 2023 Nov 22;13(1):2287119. doi: 10.1080/22221751.2023.2287119 (PMC10810669; doi:10.1080/22221751.2023.2287119)
Supplement: Supplementary_material_revised [file TEMI_A_2287119_SM7010.docx]

**Table S1.** Drug resistance pattern of 2277^†^ patients predicted by whole genome sequencing and stratified by residency status.

|  | Residents | | | Migrants | | |
| --- | --- | --- | --- | --- | --- | --- |
| Drug resistance pattern | New cases (%) | Retreated cases (%) | Total (%) | New cases (%) | Retreated cases (%) | Total (%) |
| Pan-susceptible |  |  |  |  |  |  |
| Any resistance to INH | 53 (8.2) | 8 (25.0) | 61 (8.9) | 153 (10.0) | 17 (23.6) | 170 (10.7)^*^ |
| Any resistance to RIF | 42 (6.5) | 6 (18.8) | 48 (7.1) | 109 (7.2) | 17 (23.6) | 126 (7.9)^*^ |
| Any resistance to EMB | 17 (2.6) | 5 (15.6) | 22 (3.2) | 36 (2.4) | 8 (11.1) | 44 (2.8)^*^ |
| Any resistance to PZA | 11 (1.7) | 1 (3.1) | 12 (1.8) | 30 (2.0) | 3 (4.2) | 33 (2.1)^*^ |
| MDR | 18 (2.8) | 5 (15.6) | 23 (3.4) | 62 (4.1) | 14 (19.4) | 76 (4.8)^*^ |
| MDR plus resistance to any FQs | 6 (33.3) | 0 (0) | 6 (26.1) | 15 (24.2) | 5 (35.7) | 20 (26.3)^*^ |

Abbreviations: INH, isoniazid; RIF, rifampicin; EMB, ethambutol; PZA, pyrazinamide; MDR, multidrug resistance; FQ, fluoroquinolone

^*^Compared to residents, all *P* values >0.05

^†^Residential status and treatment history was missing for 68 clinical strains wirh sequenced data

**Table S2.** Characteristics of resident cases enrolled and not enrolled in treatment in Shenzhen.

|  | Enrolled in treatment in Shenzhen | | Multivariable analysis^*^ | |
| --- | --- | --- | --- | --- |
|  | Yes (n=574) | No (n=107) | aORs (95%CI) | *P* values |
| **Demographic characteristics** | |  |  |  |
| Gender |  |  |  |  |
| Male | 368 (64.1) | 81 (75.7) | 2.18 (1.28-3.70) | 0.004 |
| Female | 206 (35.9) | 26 (24.3) | 1.00 |  |
| Age (years) |  |  |  |  |
| 15-34 | 343 (59.8) | 72 (67.3) | 1.00 |  |
| 35-54 | 200 (34.8) | 19 (17.8) | 0.56 (0.32-1.00) | 0.051 |
| 55-64 | 24 (4.2) | 12 (11.2) | 2.26 (0.99-5.18) | 0.053 |
| 65 and higher | 7 (1.2) | 4 (3.7) | 2.59 (0.69-9.79) | 0.160 |
| Occupation |  |  |  |  |
| Housekeeping/jobless | 163 (28.4) | 42 (39.3) | 1.00 |  |
| Factory workers | 238 (41.5) | 19 (17.8) | 0.38 (0.21-0.71) | 0.003 |
| Students | 11 (1.9) | 3 (2.8) | 0.97 (0.23-4.14) | 0.964 |
| Teachers or tutors | 7 (1.2) | 2 (1.9) | 1.59 (0.28-9.18) | 0.605 |
| Others | 155 (27.0) | 41 (38.3) | 1.24 (0.72-2.11) | 0.441 |
| Time to diagnosis since arrival at Longhua district (months) | | |  |  |
| ≤6 months | 38 (6.6) | 24 (22.4) | 4.39 (2.33-8.24) | <0.001 |
| >6 months | 515 (89.7) | 68 (63.6) | 1.00 |  |
| Unknown (n=186) | 21 (3.7) | 15 (14.0) | 5.63 (2.53-12.56) | <0.001 |
| **Clinical characteristics** | |  |  |  |
| Retreated cases | 27 (4.7) | 5 (4.7) | 0.79 (0.27-2.36) | 0.676 |
| Cavitary disease | 157 (27.4) | 21 (19.6) | 0.61 (0.34-1.09) | 0.096 |
| Sputum-smear positivity | |  |  |  |
| Positive | 213 (37.1) | 21 (19.6) | 1.00 |  |
| Negative | 361 (62.9) | 86 (80.4) | 2.32 (1.33-4.05) | 0.003 |
| **Bacteriological characteristics** | |  |  |  |
| Beijing strain |  |  |  |  |
| Yes | 425 (74.0) | 82 (76.6) | 1.04 (0.61-1.77) | 0.881 |
| No | 149 (26.0) | 25 (23.4) | 1.00 |  |
| Drug-resistance profile |  |  |  |  |
| MDR | 16 (2.8) | 7 (6.5) | 1.40 (0.48-4.09) | 0.541 |
| Other DR | 84 (14.6) | 17 (15.9) | 0.88 (0.47-1.66) | 0.700 |
| Pan-susceptible | 474 (82.6) | 83 (77.6) | 1.00 |  |

**Table S3.** Characteristics of migrant cases enrolled and not enrolled in treatment in Shenzhen.

|  | Enrolled in treatment in Shenzhen | | Multivariable analysis^*^ | |
| --- | --- | --- | --- | --- |
|  | Yes (n=938) | No (n=658) | aORs (95%CI) | *P* values |
| **Demographic characteristics** | |  |  |  |
| Gender |  |  |  |  |
| Male | 677 (56.6) | 520 (43.4) | 1.40 (1.08-1.80) | 0.01 |
| Female | 261 (65.4) | 138 (34.6) | 1.00 |  |
| Age (years) |  |  |  |  |
| 15-34 | 676 (72.1) | 523 (79.5) | 1.00 |  |
| 35-54 | 187 (19.9) | 94 (14.3) | 0.69 (0.51-0.92) | 0.012 |
| 55-64 | 52 (5.5) | 26 (4.0) | 0.77 (0.46-1.30) | 0.336 |
| 65 and higher | 23 (2.5) | 15 (2.3) | 0.76 (0.37-1.57) | 0.462 |
| Occupation |  |  |  |  |
| Housekeeping/jobless | 377 (40.2) | 282 (42.9) | 1.00 |  |
| Factory workers | 343 (36.6) | 211 (32.1) | 0.96 (0.75-01.23) | 0.724 |
| Students | 21 (2.2) | 24 (3.7) | 1.40 (0.74-2.63) | 0.303 |
| Teachers or tutors | 4 (0.4) | 6 (0.9) | 2.91 (0.78-10.82) | 0.111 |
| Others | 193 (20.6) | 135 (20.5) | 1.11 (0.83-1.47) | 0.493 |
| Time to diagnosis since arrival at Longhua district (months) | | |  |  |
| ≤6 months | 277 (29.5) | 284 (43.2) | 2.27 (1.81-2.86) | <0.001 |
| >6 months | 623 (66.4) | 262 (39.8) | 1.00 |  |
| Unknown (n=186) | 38 (4.1) | 112 (17.0) | 7.35 (4.91-11.02) | <0.001 |
| **Clinical characteristics** | |  |  |  |
| Retreated cases | 39 (4.2) | 33 (5.0) | 1.16 (0.69-1.94) | 0.575 |
| Cavitary disease | 245 (26.1) | 175 (26.6) | 0.99 (0.78-1.27) | 0.956 |
| Sputum-smear positivity | |  |  |  |
| Positive | 351 (37.4) | 182 (27.7) | 1.00 |  |
| Negative | 587 (62.6) | 476 (72.3) | 1.52 (1.20-1.92) | <0.001 |
| **Bacteriological characteristics** | |  |  |  |
| Beijing strain |  |  |  |  |
| Yes | 703 (75.0) | 483 (73.4) | 0.84 (0.66-1.08) | 0.172 |
| No | 235 (25.0) | 175 (26.6) | 1.00 |  |
| Drug-resistance profile |  |  |  |  |
| MDR | 45 (4.8) | 31 (4.7) | 0.99 (0.60-1.66) | 0.981 |
| Other DR | 123 (13.1) | 101 (15.4) | 1.31 (0.96-1.78) | 0.084 |
| Pan-susceptible | 770 (82.1) | 526 (79.9) | 1.00 |  |

**Figure S1.** Genealogical tree based on the time-labeled phylogenic tree of the 11 clusters with at least four patients. The star and the change of color represent the occurrence of transmission event or new infection. R, resident tuberculosis patient; M, migrant tuberculosis patient; U, untreated tuberculosis patients; T, treated tuberculosis patient.

**Figure S2.** Consensus transmission tree of the 11 clusters based on MCMC outputs by TransPhylo package analysis. Each horizontal line represents a case, and the darkness of each line point representing their changing infectivity over time. The arrows represent the occurrence of transmission event from case to case. The red circles represent the official notified time point of each case.
